# Smart Lithotripsy: Lasers, Energy Sources and Efficiency

**DOI:** 10.1007/s11934-026-01354-z

**Published:** 2026-08-17

**Authors:** Gantapong Sainont, Michael Uy, Pankaj N Maheshwari, Hsiang Ying Lee, Daniele Castellani, Yasser A. Noureldin, Brett A. Johnson, Horacio Sanguinetti, Khurshid R. Ghani, from the Office of Research FUTURE Collaborative of the Endourological Society

**Affiliations:** 1 Department of Urology, University of Michigan, Ann Arbor, MI USA; 2 Division of Urology, Department of Surgery, Faculty of Medicine, Chulalongkorn University and King Chulalongkorn Memorial Hospital, Thai Red Cross Society, Bangkok, 10330 Thailand; 3 Chief of Urology, Fortis Hospital Mulund, Mumbai, India; 4 Department of Urology, Kaohsiung Medical University Hospital, Kaohsiung Medical University, Kaohsiung, Taiwan; 5 Department of Urology, School of Medicine, College of Medicine, Kaohsiung Medical University, Kaohsiung, Taiwan; 6 Department of Medicine and Surgery, LUM University, Casamassima, Bari, Italy; 7 Urology unit, Ecclesiastical Entity Regional General Hospital “F. Miulli”, Acquaviva delle Fonti, Bari, Italy; 8 Urology Department, Northern Ontario School of Medicine University, Thunder Bay, ON Canada; 9 Department of Surgery, Sault Area Hospital, Sault Ste Marie, ON Canada; 10 Department of Urology, University of Texas Southwestern Medical Center, Dallas, TX USA; 11 Urology Department, Hospital de Clínicas, Buenos Aires, Argentina

**Keywords:** Laser lithotripsy, Smart lithotripsy, Holmium:YAG laser, Thulium fiber laser, Pulsed Thulium:YAG laser, Ureteroscopy

## Abstract

**Purpose of review:**

To examine the evidence on contemporary lasers and provide a practical framework for platform selection, parameter optimization, and thermal safety based on the principles of smart lithotripsy.

**Recent findings:**

Holmium: yttrium-aluminum-garnet (Ho:YAG) remains versatile for fragmentation and dusting, whereas thulium fiber laser (TFL) provides efficient fine-particle dusting with limited retropulsion. Pulsed thulium: YAG (p-Tm:YAG) shows an intermediate profile between Ho:YAG and TFL, but clinical evidence on lithotripsy outcomes is limited. Higher power settings with Ho:YAG may accelerate ablation, but have to be used carefully with high flow rates and access sheaths. For TFL, high-power settings have not been shown to shorten operative time or improve stone-free outcomes and may increase thermal risk. Future systems may incorporate stone recognition, composition prediction, and tissue protection.

**Summary:**

There is no ideal laser for every clinical scenario. Urologists should match the platform, settings, and treatment strategy to the intended endpoint, whether fragmentation with active extraction or dusting with passive clearance.

## Introduction

Laser lithotripsy is now one of the most common procedures performed in urology. Over the past decades, the field has evolved from rudimentary solid-state laser systems to sophisticated platforms with distinct wavelengths and mechanisms of action. However, using these lasers without a clear understanding of their capabilities and underlying principles may limit their potential. Laser–stone interaction reflects a dynamic interplay between photothermal and photomechanical effects that must be carefully managed to optimize outcomes.

This is where the concept of “smart lithotripsy” becomes relevant. Applying the same routine laser settings, or selecting a laser platform based on availability, may suffice in straightforward cases but not across all stone types and clinical scenarios. Modern day laser lithotripsy requires an understanding of the characteristics of each platform and the effects of different parameters, allowing the surgeon to tailor the technology, settings, and operative strategy to the intended surgical goal while maintaining safety. This narrative review summarizes evidence from both in vitro and clinical studies conducted within the last 5 years. Our goal is to provide a practical framework for selecting laser platforms, parameters, and strategies to optimize both efficiency and safety in modern stone surgery.

## Evolution of Laser Platforms

For decades, the Holmium:Yttrium-Aluminum-Garnet (Ho:YAG) laser has been the mainstay of endoscopic stone surgery. High-power Ho:YAG systems expanded the use of high-frequency settings and supported a broader shift toward dusting strategies during ureteroscopic lithotripsy [1, 2]. However, its high peak power inevitably generates large cavitation bubbles [3, 4], contributing to its well-recognized limitations such as stone retropulsion and suboptimal dusting quality. These shortcomings have driven development of newer platforms, including pulse-modulated Ho:YAG laser ; thulium fiber laser (TFL) [5] and more recently, pulsed Thulium: YAG (p-Tm:YAG) laser [6].

The behavior of each platform is largely defined by two key features: wavelength and pulse characteristics. Wavelength determines how strongly laser energy is absorbed by water, while pulse characteristics—including pulse shape, duration, repetition rate, and peak power—define how that energy is delivered over time. These differences help explain variations in ablation efficiency, retropulsion, and fragmentation quality.

### Key Features: Wavelength

Ho:YAG emits energy at 2100 nm, while TFL operates at 1940 nm and p-Tm:YAG at 2013 nm. At a water temperature of 20 °C, TFL and p-Tm:YAG have water absorption coefficients approximately fivefold and twofold higher than Ho:YAG, respectively [7, 8]. In theory, greater water absorption reduces laser penetration depth, potentially improving safety by limiting energy spread into surrounding tissue and altering the pattern of laser–stone interaction. At the same time, this can impact the efficiency of stone fragmentation.

### Key Features: Pulse Characteristics

**Ho:YAG laser** produces asymmetric, mode-dependent pulse profiles. Short pulse (SP) has a steep leading peak followed by a gradual decline—often described as “shark-fin” shaped—whereas long pulse (LP) has a lower initial peak, a flatter plateau, and a longer trailing slope. Pulse-modulation technologies, particularly MOSES by Boston Scientific, divide energy into two sequential sub-pulses and are available as Moses Contact (MC) and Moses Distance (MD) [9, 10]. Although current Ho:YAG systems offer a broader range of pulse durations than earlier generations, they generally retain relatively short pulses and high peak power compared with TFL, while p-Tm: YAG characteristics are in between TFL and Ho:YAG laser (Table 1) [9–13].

**TFL** produces a uniform, rectangular flat-top pulse profile [4, 9, 14]. Evolving from continuous-wave (CW) systems to the current Superpulse platform, TFL (IPG Medical) can operate in both quasi-CW and pulsed modes. Compared with Ho:YAG laser, TFL delivers substantially longer pulses at a lower, more stable peak power (Table 1) [9, 15, 16] and produces cavitation bubbles approximately four times smaller than those of Ho:YAG [4].

**P-Tm:YAG laser** is currently available in two main systems: Thulio (Dornier Medtech, Germany) and RevoLix HTL (Omniguide, USA) [6]. Although both produce near-rectangular pulse profiles, their pulse duration and peak power differ substantially (Table 1). Measured values place RevoLix HTL closer to TFL than to Thulio, likely reflecting its hybrid pulsed/CW design [6, 17–20].

### Strategy in Smart Lithotripsy

**Fig. 1:**
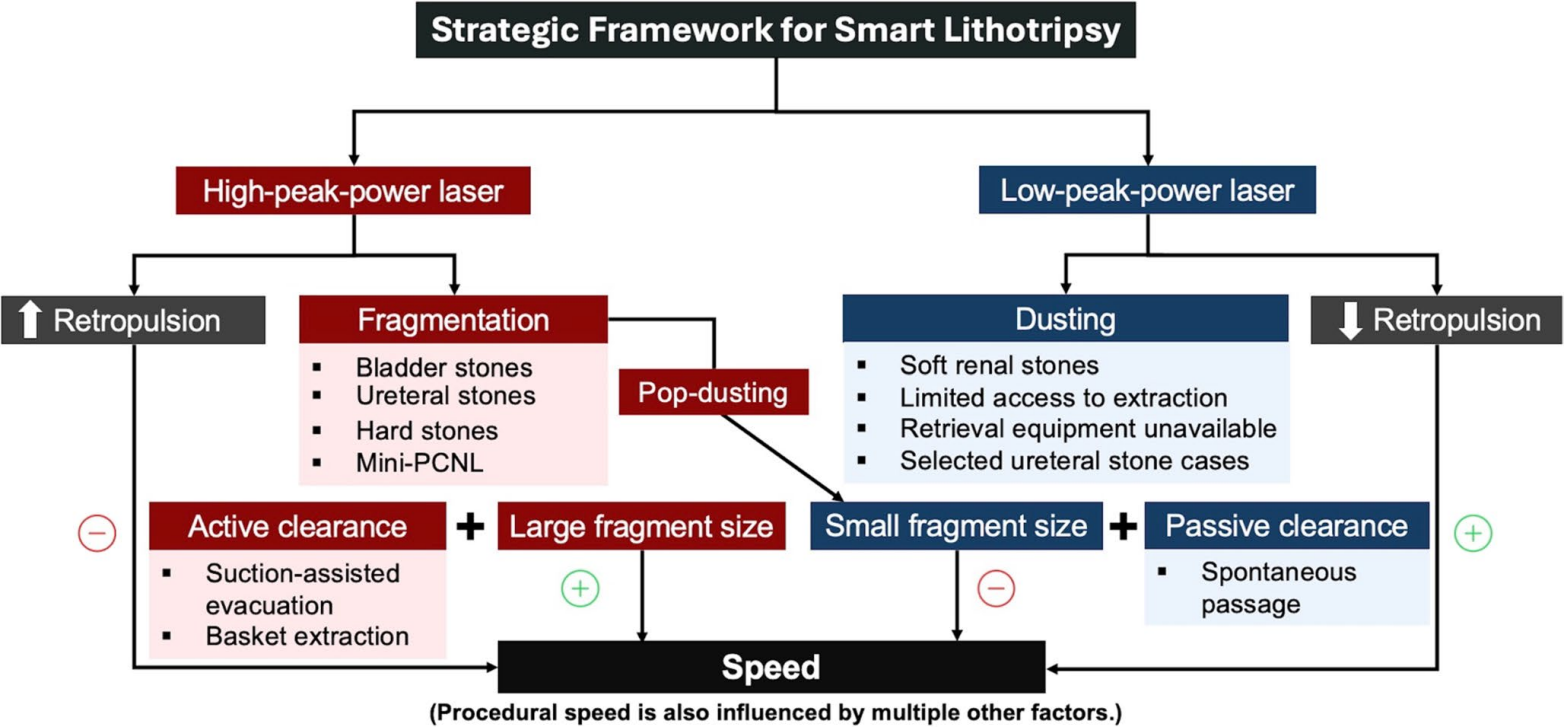
Strategic framework for selecting a smart lithotripsy approach. High-peak-power lasers favor fragmentation into larger pieces, followed by active clearance using basket extraction or suction-assisted evacuation. This approach can be particularly efficient for bladder stones, ureteral stones, hard stones, and mini-PCNL. High-peak-power lasers are excellent for the pop-dusting technique to further reduce residual fragments to a size suitable for passive clearance. Low-peak-power lasers favor dusting into smaller particles with less retropulsion and are preferred for soft renal stones, limited access to extraction, unavailable retrieval equipment, or selected ureteral stones. The resulting particles may pass spontaneously or be aspirated using FANS or direct in-scope suction. + and − indicate factors that may increase or decrease procedural speed, respectively. Actual performance also depends on stone, anatomical, technical, irrigation, and suction-related factors. Abbreviations: PCNL, percutaneous nephrolithotomy; FANS, flexible and navigable suction ureteral access sheath

A smart lithotripsy conceptual framework should begin with the desired lithotripsy endpoint: do you want fine particles for passive clearance or into retrievable fragments for active extraction or suction? High peak-power lasers such as Ho:YAG break stones rapidly into larger fragments but generate significant retropulsion. Low peak-power lasers such as TFL produce finer dust; while ablation may appear slower initially, reduced retropulsion keeps the stone stable and minimizes repeated repositioning, which can improve efficiency. This conceptual framework divides laser lithotripsy into two distinct strategies: 1) High Peak Power / Large Fragment Size with Active Clearance; 2) Low Peak Power / Low Fragment Size with Dusting and Passive Clearance. Figure 1 details this.

### Platform-Specific Strategies: Thulium Fiber Laser

The switch from Ho:YAG to TFL platforms has created a new challenge in clinical practice. Because TFL has distinct physical properties, its settings should not simply follow the traditional Ho:YAG concept of “low energy and high frequency” for dusting. With a growing body of literature, several principles can now guide parameter selection.

### Pulse duration

SP settings consistently yield greater ablation than LP modes in bench studies. Soto-Palou et al. found that SP produced significantly greater stone ablation at all tested standoff distances in an automated dusting model [21], consistent with the single-pulse analysis by Marom et al., who showed that SP created wider and deeper ablation craters than those produced with LP [14]. Johnson et al. similarly found that inadequate ablation occurred more often with medium- or LP settings across seven human stone compositions, while SP was generally favored at practical settings and also reduced charring and sparking [22]. Based on these findings, SP can be considered the default mode for most TFL lithotripsy applications. It is important to note that SP mode in TFL is not directly comparable to SP mode in Ho:YAG, as TFL pulse durations are very long, considerably longer than the longest Ho:YAG pulses (see Table 1).

**Table 1:** Technical characteristics and pulse parameters of contemporary laser lithotripsy platforms

Parameter	Ho: YAG	TFL	*p*-Tm:YAG
Pumping Source	Flashlamp	Laser diodes	Laser diodes
Active Medium	Solid-state crystal	Doped silica fiber	Solid-state crystal
Wavelength	2100 nm	1940 nm	2013 nm
Pulse Shape	Asymmetric, mode-dependent; typically shark-fin shaped	Near-rectangular	Near-rectangular
Pulse Duration	150–1,500 µs (SP: ~140–350 µs) [9, 11–13] MC: first/second sub-pulses, 100/285 µs (total duration, 385 µs) [10] MD: first sub-pulses, 200 µs; interval, 100 µs; second sub-pulse, 240 µs (total duration, 540 µs) [10]	400–50,000 µs [9, 15, 16]	**Thulio**: 150–1,200 µs (spec.) [19]; ~370–1,200 µs (measured) [17] **RevoLix HTL**: 200–4,750 µs (spec.) [20]; ~1,102–3,044 µs (measured) [18]
Peak Power	SP: ~2,800 to >13,000 W LP/Moses: ~1,800–6,100 W [9, 10]	125–550 W [9, 15, 16]	**Thulio**: max ~ 3,700 W (spec.) [19]; mean ~500–2,200 W (measured) [17] **RevoLix HTL**: max ~ 1,300 W (spec.) [20]; ~325–565 W (measured) [18]

**Abbreviations**: *Ho:YAG* holmium:yttrium-aluminum-garnet laser, *TFL* thulium fiber laser, *p-Tm:YAG* pulsed thulium:yttrium-aluminum-garnet laser, *SP* short pulse, *LP* long pulse, *MC* Moses Contact, *MD* Moses Distance, *spec*. manufacturer specifications, *max* maximum, *nm* nanometers, *µs* microseconds, *W* watts

### Pulse Energy and Frequency

The optimal balance between pulse energy (Ep) and frequency (F) with TFL remains debated. Although increasing either parameter can improve ablation efficiency when the other is held constant [23], the more clinically relevant question is which Ep/F combination performs best at equivalent total power. Bench studies have provided conflicting answers, underscoring the need for caution when interpreting experimental lithotripsy data, which do not always convert into the same outcome in clinical practice. These may be influenced by several factors, including artificial stone composition, fiber-control method, laser platform, irrigation conditions, and working distance. Among these, retropulsion is important, as it can alter ablation performance depending on whether stones are fixed or free to move. In fixed-stone models, the findings are relatively consistent. **At equivalent total power**,** high-Ep/low-F settings generally demonstrate greater stone ablation than low-Ep/high-F settings across most tested conditions** [21, 23–25]. This effect appears to be driven mainly by enlargement of the ablation crater area, whereas changes in crater depth are relatively limited [14]. Within the low-power range of ≤10 W, settings such as 0.8 J/12 Hz and 1.0 J/10 Hz have shown high efficiency [21, 24, 26].

In contrast, findings from mobile-stone models are less uniform. Mishra et al. and Liao et al., using artificial stones in a kidney model and experimental cuvettes, respectively, reported results broadly consistent with fixed-stone studies: high-Ep/low-F settings produced greater ablation efficiency than low-Ep/high-F settings, with efficiency increasing almost linearly as Ep increased [26, 27]. However, Zha et al. observed a different pattern using a model in which stones were tested in a long horizontal glass tube and fiber–stone contact had to be repeatedly re-established after displacement. In that setting, the best balance between mass loss and retropulsion occurred at 0.3–0.5 J and 20–100 Hz, whereas pulse energies >1 J reduced ablation efficiency and increased retropulsion; frequencies >100 Hz also impaired efficiency, although this effect was less pronounced [28]. Johnson et al. further highlighted this in a study of human stone subtypes where relatively low-Ep/high-F settings tended to provide favorable ablation efficiency for most stones [22].

### Total power

Among the parameters discussed so far, total power shows the most consistent bench-study trend: higher power generally increases ablation rate [22, 24, 26, 28]. An interesting study by Zha et al. compared settings of 10, 20, and 30 W. There was greater ablation with 20 W vs. 10 W settings, but when 30 W was assessed, the differential increase was not as substantial. For example, the ablation amount, measured as mass loss, was 10.0, 25.2, and 30.6 mg, comparing 1 Jx 10 Hz (10 W), 1 Jx 20 Hz (20 W) and 1 Jx 30 Hz (30 W), respectively [28]. With TFL, higher power does not necessarily translate into better clinical performance. In a randomized trial by Æsøy et al., sheathless ureteroscopic treatment of 8–25 mm renal stones was performed using high-power TFL (16–18 W; 0.4 J/40 Hz, 0.6 J/30 Hz, or 0.8 J/20 Hz) or low-power TFL (4–6 W; 0.4–0.6 J at 10 Hz). Although high-power TFL produced nearly twice the ablation speed, operative time was not significantly reduced, with only an approximately four-minute difference between groups. In contrast, low-power TFL achieved higher rates of zero residual fragments (63% vs. 44%) and residual fragments <2 mm on three-month NCCT (77% vs. 56%) [29]. Laser-use patterns may partly explain this finding: Low-power was associated with longer active laser time and higher operator duty cycle (ODC), while total laser operating time was similar between groups [30]. This suggests that low-power setting allowed more sustained and continuous lasing, whereas high-power setting was more likely to require intermittent activation because of retropulsion, thermal concerns, and impaired visibility from the snowstorm effect, particularly in a sheathless setting.

**Fiber-to-stone distance** is critical for TFL efficiency. Maximum ablation occurs at near-stone contact (~0.2 mm) [21], whereas efficiency drops markedly, with nearly no ablation when the laser fiber is at distances of ~1 mm or greater [14, 23]. Therefore, TFL should be used in contact or near-contact with a controlled painting motion. Overall, TFL appears inherently suited to a dusting strategy, producing fine particles across a range of settings without requiring high power. Because these fragments are intended to clear passively in this strategy, the added clinical value of adjunctive technologies such as flexible and navigable suction ureteral access sheath (FANS) or direct in-scope suction requires further studies.

### Platform-Specific Strategies: Holmium:YAG laser

Ho:YAG laser remains highly versatile and adaptable for both fragmentation and dusting strategies. Although a fragmentation approach has become less dominant in the modern era of dusting, it remains an effective strategy when used in appropriate situations (see Fig. 1). The recommended approach is a high-Ep/low-F setting, typically in the range of 0.6–1.2 J at 6–10 Hz [31]. Starting with a modest Ep and escalating only when necessary, may help preserve the fiber tip while minimizing avoidable retropulsion.

For dusting, an advantage of Ho:YAG laser over TFL is its ability to deliver energy at a greater distance from the fiber tip, reflecting its lower water absorption and greater optical penetration depth. For this reason, dusting can be performed using either contact or non-contact techniques. In terms of settings, low-Ep/high-F combinations (e.g., 0.2–0.5 J at 20–80 Hz) paired with a long pulse duration have consistently been shown to optimize dusting [2, 32] while minimizing retropulsion and fiber burnback. Nevertheless, the in vitro study by Aldoukhi et al. demonstrated a threshold effect in Ho:YAG laser dusting, where increasing frequency improved ablation only up to a certain point before reaching a plateau. This was attributed to reduced energy delivery to the crater base, particularly when the laser fiber was held in a fixed position. The same study also showed that the benefit of higher frequency became more pronounced as the speed of fiber movement increased [33]. This supports a continuous “painting” or “dancing” technique across the stone surface, rather than keeping the fiber stationary, to maintain consistent and efficient energy delivery [34].

When multiple small fragments remain after contact dusting, non-contact lithotripsy or “popcorning” or “pop-dusting” can serve as a finishing step to reduce fragment size. Current evidence does not define a single optimal setting, but a reasonable practical range for Ho:YAG laser includes 1 J/20 Hz, 1.5 J/20 Hz, or 0.5 J/40–80 Hz [35–38]. Aldoukhi et al. showed that popcorning achieved a finer fragment distribution when the fiber was kept in contact with the stone collection rather than 2 mm away, and when performed in a smaller calyceal model. Accordingly, “non-contact” should not imply positioning the fiber far from the stones; the fiber tip should remain close to the collection of stone fragments within a confined calyx while avoiding urothelial injury [35]. For pulse mode, available data are mixed: LP was favored in some early studies [39], whereas Aldoukhi et al. found SP superior at a 2-mm working distance [35]. For pulse modulation such as Moses technology, bench tests showed that Moses modes produced greater stone ablation than regular mode [40]. In pop-dusting models, MC performed better in producing very fine fragments ≤0.5 mm when the fiber tip was positioned within the stone fragments [41]. However, MD mode appeared to provide the greatest overall stone ablation across different fiber-to-stone positions [10, 38, 41, 42], whether the fiber was close to the stone or positioned farther away. This may make MD a practical option for pop-dusting.

In contrast to TFL, clinical evidence with Ho:YAG laser does not support a power threshold such as 20 W. Several studies using CT to assess stone-free rates have shown that high-power pop-dusting ranging from 40 W to 100 W settings can shorten stone-disintegration time or operative time with excellent stone-free rates and no increase in complications [43–45] (Table 2). In all these studies, clinical parameters required the use of access sheaths with high-flow irrigation to reduce concerns around heat generation (see later). High-power Ho:YAG may therefore be used effectively when paired with an appropriate technique and an operative environment that provides sufficient irrigation, and is now increasingly attractive with the emergence of FANS which allows higher flow combined with suction, which will translate into a decrease in intrarenal pressure and heat generation inside the kidney.

**Table 2:** CT-based stone-free rates after high-power Ho:YAG lithotripsy for retrograde intrarenal surgery

Study	Study design	Ho:YAG power	Stone-free rate	Comments
Chung et al., [43]	Retrospective, single-center (*n* = 82)	Pop-dusting ~ 100 W	89%	Complete SFR assessed at 3 months Mean OT 58.1 min Patients also underwent MAG3 scans No strictures, hematoma or renal function deterioration
Peretti et al., [44]	Retrospective case-control (*n* = 199)	High-power (120 W laser): pop-dusting at 40 W vs. Lower-power (30 W laser): popcorning at 15 W	69% (high-power)	Complete SFR at 3 months SFR with lower-power 65%; higher composite success (93%) with high-power vs. low-power (82%) Shorter OT with higher-power 56.6 vs. 65.2 min
Inoue et al., [45]	Retrospective, single-center (*n* = 305)	Fragmentation (0.6–1.2 J × 6–10 Hz) followed by 40-W Moses pop-dusting	75.1%	Complete SFR (no residual stones) assessed by CT at 1 month Lower pole stones only cohort

**Abbreviations**: *CT* computed tomography, *Ho:YAG* holmium:yttrium-aluminum-garnet, *Hz* hertz, *J* joule, *MAG3* mercaptoacetyltriglycine, *OT* operative time, *SFR* stone-free rate, *W* watt

### Pulse Modulation platforms

Pulse modulation has become one of the key innovations allowing the Ho:YAG laser to remain competitive with newer platforms. MOSES technology by Boston Scientific was the first widely adopted pulse-modulation system, followed by Virtual Basket by Quanta System, both of which work by splitting the laser emission into two sequential sub-pulses, where energy is delivered to the target by the second sub-pulse.

The newest addition is Magneto technology by Quanta System, which takes pulse modulation a step further by prolonging pulse duration to approximately 2000 µs and lowering peak power to around 500 W — a profile designed to mimic the dusting behavior of TFL. However, detailed pulse characteristics have not yet been described in the published literature. The only available bench study, by Folcia et al., compared Magneto Ho:YAG laser with TFL at an equal total power of 10 W and found slightly shorter retropulsion distances with Magneto at low pulse energies of 0.2 and 0.4 J, but no difference at higher pulse energies of 0.8, 1.5, and 2 J [46]. Evidence at this point is limited, and further studies are needed to define Magneto’s role among pulse-modulation options.

### Pulsed-Thulium:YAG laser

Pulsed-Thulium:YAG has emerged as a platform that may bridge the gap between Ho:YAG laser and TFL. Its pulse profile and peak-power characteristics sit closer to TFL, producing an elongated vapor bubble rather than the broad, spherical bubble typical of Ho:YAG laser [47]. However, because p-Tm:YAG has only recently entered clinical use for stone surgery, evidence is limited.

Early bench data support its dusting capability. Kwok et al. showed that p-Tm:YAG could produce fine dust particles < 250 μm across seven human stone compositions using different Ep/F combinations, including 0.1 J/100 Hz, 0.4 J/25 Hz, and 2 J/5 Hz, reaching also particles of 63 μm [19]. In laboratory retropulsion testing, p-Tm:YAG also produced significantly less retropulsion than Ho:YAG laser, supporting its potential advantage for dusting [48].

Clinical evidence is still lacking. Comparisons between p-Tm:YAG laser and TFL from a non-randomized prospective study [49] and a multicenter study have produced inconsistent findings. Operative time has generally been similar, but outcomes such as lasing time and zero-fragment rate have varied. In the study by Castellani et al., p-Tm:YAG laser achieved a higher zero-fragment rate on 30-day noncontrast CT compared with TFL (71.8% vs. 62.5%, *p* = 0.04). However, this was accompanied by a higher reintervention rate (17.2% vs. 3.1%) and a greater proportion of 2–4 mm residual fragments at 30 days (14.1% vs. 6.3%) [50]. These discordant findings raise the question of whether p-Tm:YAG dusting performance in real-world practice is truly comparable to TFL. In a randomized trial including both ureteral and renal stones, p-Tm:YAG laser showed similar operative time and 6-week stone-free rates compared with the high-power Ho:YAG laser using a <2 mm threshold (65.1% vs. 62.5%) [51]. Median energy use was slightly lower with p-Tm:YAG laser, but this difference was not statistically significant (4.71 vs. 5.31 kJ) [51]. Overall, p-Tm:YAG laser appears promising, but its clinical role requires further studies to define optimal settings and advantages.

### Ho:YAG vs. TFL vs. p-Tm:YAG

Direct in vitro comparisons help clarify the differences among contemporary platforms. In a standardized robotic model using synthetic stones, TFL generally produced the highest volumetric ablation performance, with p-Tm:YAG showing intermediate results and Ho:YAG laser the lowest overall. However, the gap between TFL and the p-Tm:YAG laser was not uniform across all conditions; p-Tm:YAG remained competitive in several settings and appeared particularly promising for hard-stone fragmentation [52]. A similar pattern was observed in a ureteral stone model by Sierra et al., in which p-Tm:YAG laser and TFL showed comparable ablation efficiency and speed, with both outperforming Ho:YAG laser under the tested conditions [53].

In vitro studies using human stones suggest that platform performance is strongly composition-dependent. In studies using calcium oxalate monohydrate (COM), uric acid (UA), and cystine (CYS) stones, TFL tended to perform best for COM, or at least showed favorable energy efficiency for this stone type. In contrast, Ho:YAG laser showed an advantage for UA and CYS in several comparisons, particularly when ablation volume per pulse and required energy were considered. p-Tm:YAG laser generally showed an intermediate profile, without a consistent advantage over either TFL or Ho:YAG laser across all compositions [54, 55]. These findings caution against assuming that any single platform is universally superior.

### Thermal Safety in Smart Lithotripsy

Smart lithotripsy is not only about maximizing stone ablation efficiency but also about keeping in mind thermal safety. Thermal injury depends on both the magnitude of temperature rise and the duration of tissue exposure, making thermal dose more clinically meaningful than peak temperature alone. Three modifiable laser-related factors determine heat generation: total power, activation duration, and operator duty cycle. Non-laser factors that influence heat generation also include the flow rate and baseline temperature of the irrigation fluid.

**Total power** has a direct relationship with thermal risk. Bench studies consistently show that higher power produces higher peak temperatures and shortens the time to reach thermal injury thresholds [56, 57]. Based largely on in vitro and animal data, practical upper limits of 10 W in the ureter and 20 W in the kidney have been proposed for TFL with irrigation, with slightly higher thresholds suggested for Ho:YAG laser in flexible ureteroscopy [57–59]. Gupta et al. provide important human data by measuring real-time calyceal fluid and renal parenchymal temperatures during 2-minute testing of each Ho:YAG and TFL mode in patients undergoing ureteroscopy. Gravity irrigation with room-temperature saline was delivered from a bag positioned 80 cm above the patient’s abdomen; the actual flow rate was not reported, but in such a scenario, it can be <10 ml/min. Under these conditions, low-power fragmentation at 2–2.5 W kept calyceal and parenchymal temperatures below 41 °C, whereas 15 W dusting exceeded 43 °C in some patients, and 40 W pop-dusting (0.5 J/80 Hz) frequently exceeded this threshold in both calyceal fluid and renal parenchyma, regardless of laser platform [60]. These findings support that higher-power settings (20–40 W) require irrigation flow beyond that provided by gravity-based systems to improve thermal safety.

**Activation duration** independently influences thermal dose. Real-world laser-log data show a mean pedal activation time of approximately 3.6 s, while longer activations increase peak temperature and thermal dose even when total energy and ODC are equivalent [61]. Under the same gravity-irrigation conditions, 40-W pop-dusting at 50% ODC exceeded 43 °C within 15 s, whereas shorter activation cycles appeared safer [60]. Accordingly, short, intermittent firing—approximately 5–10 s per activation—is recommended, while continuous lasing beyond 15–20 s should be avoided, particularly at high power or with limited irrigation or outflow [60–62].

**ODC** (namely the percentage of time the practitioner is actively firing the laser) is the final concept that links power and activation time. It represents the proportion of lithotripsy time during which the laser is actively firing; a higher ODC leaves less time for cooling between activations [61, 63]. In a 30-W Ho:YAG model, ODC values of 70% or higher exceeded the thermal injury threshold, while reducing ODC from 60% to 50% markedly prolonged the time to reach this threshold [63]. However, ODC should be interpreted in the context of power. In a clinical trial of TFL lithotripsy, low-power settings allowed longer activations and a higher ODC, whereas high-power settings produced shorter bursts with more frequent pauses [30]. In practice, low-power lithotripsy may tolerate a higher ODC, while high-power lithotripsy requires more deliberate pauses.

### Future Directions of Smart Lithotripsy

The future of smart lithotripsy will likely move from fixed laser settings toward feedback-guided treatment. One early step is stone recognition. Zhu et al. used a machine-learning model trained on intraoperative endoscopic images to predict stone composition, suggesting that future systems may tailor laser settings by stone type rather than surgeon experience alone [64]. A recent systematic review from the EAU Endourology group identified navigation, stone–tissue differentiation, and stone composition classification as key areas for intraoperative AI in endoscopic lithotripsy, although most evidence remains experimental and requires clinical validation [65].

Real-time safety control is another important direction. Korneva et al. applied optical feedback during laser lithotripsy in 22 patients and used machine-learning models to distinguish stone from soft tissue, with approximately 93% performance for both targets [66]. Traxer et al. also reported a preclinical TFL system that adjusted laser emission based on real-time stone recognition. The system analyzed the spectral signature of light reflected from the target through the laser fiber, permitting emission only when a stone was detected and automatically suspending emission when the fiber faced tissue or was beyond the predefined working distance. This reduced off-target tissue exposure while preserving dusting efficiency [67]. These studies suggest that future lithotripsy will increasingly focus on intelligent energy delivery, integrating stone recognition, tissue protection, temperature control, and laser-use metrics to improve efficiency while maintaining safety.

## Conclusions

Contemporary laser lithotripsy comprises three principal platforms – Ho:YAG laser, TFL, and p-Tm:YAG laser – each with distinct physical and clinical characteristics, with no single platform consistently superior across all scenarios. Smart lithotripsy requires matching the platform, settings, and treatment strategy to the stone, the patient’s anatomy, and the operative environment. The desired fragment endpoint provides a practical starting point: fragmentation with active retrieval for bladder stones, distal ureteral stones, and mini-PCNL, and dusting with passive clearance for soft renal stones or when extraction is not desired or feasible. TFL offers inherent advantages for dusting through its low peak power and reduced retropulsion, whereas the Ho:YAG laser remains versatile across both strategies and has been enhanced by pulse modulation. The p-Tm:YAG laser shows early promise, although its clinical role requires further prospective evaluation. Thermal safety must remain integral to treatment planning, with deliberate management of power, activation duration, and ODC. As feedback-guided and AI-assisted systems enable stone recognition, composition prediction, and real-time tissue protection, these principles will remain essential for precise, efficient, and safe patient-tailored stone surgery.

## Key References

- Æsøy MS, Juliebø-Jones P, Gjengstø P, Beisland C, Ulvik Ø. Watt’s the difference? A randomised trial of high- vs low-power ureteroscopic thulium fibre laser lithotripsy. BJU Int. 2026;137(3):485–492. 10.1111/bju.70123.
  - ○ A randomized trial showing that higher TFL power increased ablation speed without improving operative time or stone-free outcomes.
- Zha R, Wang C, Liao Y, Li Z, Li S, Wang H, et al. Optimal thulium fiber laser parameters for non-impacted stone lithotripsy: an in-vitro and ex-vivo evaluation. Urolithiasis. 2026;54(1).
  - ○ An in vitro and ex vivo study exploring TFL ablation across different power settings and demonstrating a 20-W threshold, with diminishing gains at higher power levels.
- Traxer O, Zorzi F, Yaroslavsky IV, Kovalenko A, Andreeva V, Childs J, et al. Pre-clinical evaluation of a laser system with emission control based on stone vs soft tissue recognition. BJU Int. 2026. 10.1111/bju.70301.
  - ○ A preclinical study of real-time stone–tissue recognition and automatic laser emission control.

## Data Availability

No datasets were generated or analysed during the current study.
